# A tRNA-Acetylating Toxin and Detoxifying Enzyme in Mycobacterium tuberculosis

**DOI:** 10.1128/spectrum.00580-22

**Published:** 2022-05-31

**Authors:** Francesca G. Tomasi, Alexander M. J. Hall, Jessica T. P. Schweber, Charles L. Dulberger, Kerry McGowen, Qingyun Liu, Sarah M. Fortune, Sophie Helaine, Eric J. Rubin

**Affiliations:** a Department of Immunology and Infectious Diseases, Harvard T. H. Chan School of Public Health, Boston, Massachusetts, USA; b Department of Microbiology, Harvard Medical School, Boston, Massachusetts, USA; University of Manitoba

**Keywords:** *Mycobacterium tuberculosis*, antibiotic resistance, tRNA modification, toxin/antitoxin systems, translation

## Abstract

Toxin-antitoxin (TA) systems allow bacteria to adapt to changing environments without altering gene expression. Despite being overrepresented in Mycobacterium tuberculosis, their physiological roles remain elusive. We describe a TA system in M. tuberculosis which we named TacAT due to its homology to previously discovered systems in Salmonella. The toxin, TacT, blocks growth by acetylating glycyl-tRNAs and inhibiting translation. Its effects are reversed by the enzyme peptidyl tRNA hydrolase (Pth), which also cleaves peptidyl tRNAs that are prematurely released from stalled ribosomes. Pth is essential in most bacteria and thereby has been proposed as a promising drug target for complex pathogens like M. tuberculosis. Transposon sequencing data suggest that the *tacAT* operon is nonessential for M. tuberculosis growth *in vitro*, and premature stop mutations in this TA system present in some clinical isolates suggest that it is also dispensable *in vivo*. We assessed whether TacT modulates *pth* essentiality in M. tuberculosis because drugs targeting Pth might prompt resistance if TacAT is disrupted. We show that *pth* essentiality is unaffected by the absence of *tacAT*. These results highlight a fundamental aspect of mycobacterial biology and indicate that Pth’s essential role hinges on its peptidyl-tRNA hydrolase activity. Our work underscores Pth’s potential as a viable target for new antibiotics.

**IMPORTANCE** The global rise in antibiotic-resistant tuberculosis has prompted an urgent search for new drugs. Toxin-antitoxin (TA) systems allow bacteria to adapt rapidly to environmental changes, and Mycobacterium tuberculosis encodes more TA systems than any known pathogen. We have characterized a new TA system in M. tuberculosis: the toxin, TacT, acetylates charged tRNA to block protein synthesis. TacT's effects are reversed by the essential bacterial enzyme peptidyl tRNA hydrolase (Pth), which is currently being explored as an antibiotic target. Pth also cleaves peptidyl tRNAs that are prematurely released from stalled ribosomes. We assessed whether TacT modulates *pth* essentiality in M. tuberculosis because drugs targeting Pth might prompt resistance if TacT is disrupted. We show that *pth* essentiality is unaffected by the absence of this TA system, indicating that Pth's essential role hinges on its peptidyl-tRNA hydrolase activity. Our work underscores Pth's potential as a viable target for new antibiotics.

## INTRODUCTION

Mycobacterium tuberculosis, which causes tuberculosis (TB), is a leading cause of global infectious disease mortality (1). The ability of M. tuberculosis to regulate its growth in different stressful conditions *in vitro* is thought to be an important part of its success *in vivo*. One of this pathogen’s tools for growth regulation is an expansive network of toxin-antitoxin (TA) systems, with at least 100 putative modules that encompass nearly 4% of M. tuberculosis’s coding capacity (2, 3). Most of these systems in M. tuberculosis can be grouped into five main families based on sequence homology: VapBC, MazEF, RelBE, HigBA, and ParDE (4, 5). Toxins of TAs are characterized by their general intracellular targets and mechanisms of activity with most known M. tuberculosis toxins being RNases that cleave rRNA, mRNA, or tRNA.

In type II TA systems, the most widespread and heavily studied type, a protein antitoxin is bound tightly to its cognate protein toxin and acts to neutralize it (6). If the antitoxin is degraded, the toxin assumes its active form and blocks an essential process such as DNA replication or protein synthesis until antitoxin production resumes (6). Despite being widespread in bacteria, the physiological roles of TA systems are just emerging, with some having been linked to plasmid maintenance, bacteriophage immunity, and the formation of dormant, antibiotic-tolerant persisters (7, 8). TA systems might play a role in M. tuberculosis’s ability to withstand host and antibiotic pressures by controlling growth under different stress conditions (4, 9, 10). However, it remains to be determined whether or to what extent they play a role in pathogenesis. A significant barrier to understanding TA systems is the challenge of directly measuring native toxin activity in cells and, therefore, understanding when they are active and how they interact with other enzymes. Because of the nature of TA system autoregulation and posttranslational control, transcription upregulation data alone do not necessarily indicate toxin activation (11). So far, studies investigating TA systems in bacteria often measure activity in cells using ectopic overexpression constructs (4, 5, 12). These studies offer fascinating mechanistic insights but do so in isolation from other intracellular systems, and it has been difficult to link the molecular mechanisms of TA systems to their biological roles.

Recently, a new class of TA systems called TacAT was discovered in Salmonella and homologs have since been identified in other species, including Escherichia coli and Klebsiella pneumoniae (13–17). The toxins in this family are GCN5-related N-acetyltransferases (GNAT) that acetylate aminoacyl tRNA and block the incorporation of an amino acid into a growing peptide chain. TacT’s unique mechanism of action – which can be detected using liquid chromatography-coupled mass spectrometry (LCMS) – makes it an appealing TA system to study in the context of bacterial physiology (13, 14, 18, 19). An unusual aspect of TacAT systems is that, while the antitoxin can block toxin activity as seen with other TA systems, the effect can also be reversed via the ubiquitous and essential bacterial enzyme peptidyl tRNA hydrolase (Pth) (13, 14). This enzyme cleaves acetylated amino acids from tRNA molecules, effectively unblocking protein synthesis. TacT’s mechanistic connection to an essential enzyme makes it an appealing TA system to study in the context of gene essentiality.

Here, we describe an M. tuberculosis homolog of the TacAT TA system, the first of its kind to be identified in this organism. We show that this TA system is encoded by the Rv0918-0919 operon and confirm that Rv0919 encodes a tRNA-acetylating toxin whose activity can be reversed by M. tuberculosis Pth (Rv1014c). While *pth* is required for growth in M. tuberculosis, transposon sequencing data suggest that the M. tuberculosis TacAT operon is dispensable for growth *in vitro*, and we have identified premature stop mutations in this TA system in clinical isolates (20). If TacT activity modulates *pth* essentiality in M. tuberculosis, then drugs targeting Pth might prompt resistance if TacAT activity is disrupted as has already happened in clinical isolates. However, we found that while the *tacAT* operon is indeed dispensable, *pth* essentiality is unaffected by the absence of this TA system. Our results indicate that Pth’s essential role in M. tuberculosis hinges on its function in cleaving peptidyl-tRNA and not acetylated aminoacyl tRNA. Our work underscores Pth’s potential as a viable target for new antibiotics, while also highlighting multiple angles from which to study TA systems in M. tuberculosis.

## RESULTS

### Rv0918-0919 encodes a toxin-antitoxin system that inhibits growth by acetylating glycyl-tRNAs.

**FIG 1:**
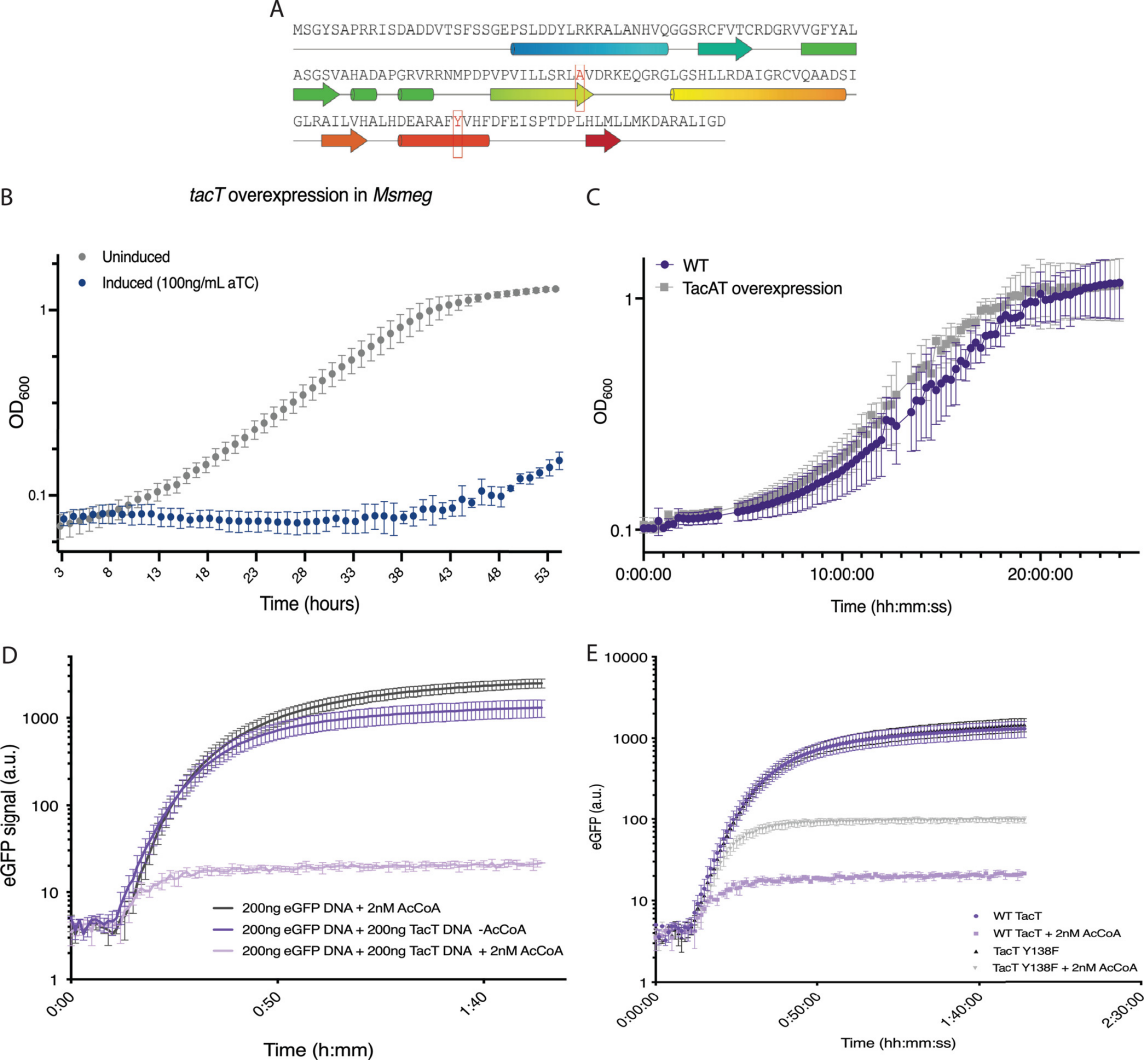
TacT is a toxin that inhibits protein synthesis. (A) Amino acid sequence and high-confidence model of M. tuberculosis TacT (Rv0919) generated using the PHYRE2 protein fold recognition server (46), with the final output modeled on the structure of TacT1 from Salmonella Typhimurium (PDB: 5fvj) (14). Active site residues in red were mutated for catalytically inactive TacT for subsequent experiments. (B) Exogenous overexpression of TacT (Rv0919) blocks the growth of Mycobacterium smegmatis (*Msmeg*). The M. tuberculosis *tacT* gene was cloned under a tetracycline-inducible promoter and integrated into the M. smegmatis genome. The addition of anhydrous tetracycline (aTC; 100 ng/mL) leads to overexpression of *tacT*. Strains were diluted to an OD of 0.003 and growth was measured by optical density at 600 nm (OD_600_). Results are shown from 3 biological replicates. (C) Exogenous expression of M. tuberculosis TacAT (Rv0918-0919) does not block the growth of Mycobacterium smegmatis. The M. tuberculosis *tacAT* operon was cloned under a constitutive overexpression promoter and integrated into the M. smegmatis genome. Strains were diluted to an OD of 0.05 and growth was measured by optical density at 600 nm (OD_600_). Results are from three replicates. (D) M. tuberculosis TacT blocks translation in the presence of acetyl coenzyme A. A TacT expression construct was added to the PURExpress *in vitro* Protein Synthesis kit along with an eGFP expression construct. Protein synthesis was read out as eGFP synthesis and monitored spectrophotometrically at excitation of 488 nm and emission of 509 nm. Results are from technical duplicates. (E) The active site mutation Y138F based on studies in Salmonella reduces M. tuberculosis TacT toxicity. Cell-free protein synthesis reactions were carried out as in (D). Inactive TacT was made using an Rv0919 expression construct containing the active side residue mutation Y138F. Results are from technical duplicates.

Previous studies have identified over 100 putative TA systems in M. tuberculosis, based on genetic architecture and homology to known TA systems (5). The M. tuberculosis operon Rv0918-0919 has been computationally flagged as a possible TA system due to its polycistronic organization and the presence of a conserved, DNA-binding ribbon-helix-helix (RHH) domain in the putative antitoxin gene, Rv0918 (Fig. 1A) (5, 21). Rv0919 contains a conserved GNAT domain, and protein BLAST results show >50% sequence identity to the N-acetyltransferase TacAT toxins in Salmonella (Fig. 1A) (22).

We hypothesized that Rv0919 encodes a TacT-like toxin that inhibits growth by blocking translation. The closely related but faster-growing, nonpathogenic model organism Mycobacterium smegmatis (*Msmeg*) does not encode any putative TacAT-like systems (21, 22). Therefore, to study M. tuberculosis TacT in isolation from other potential interacting genes, we built an integrating vector carrying Rv0919 under the control of an anhydrous tetracycline (aTC)-inducible promoter. Induced overexpression of Rv0919 in M. smegmatis inhibited growth (Fig. 1B), while constitutive expression of the entire Rv0918-0919 operon did not (Fig. 1C), showing that Rv0919 encodes a growth-inhibiting enzyme that is not active in the presence of Rv0918.

We next assessed Rv0919 activity *in vitro* using a cell-free protein synthesis kit. We found that, while GFP could be efficiently expressed in this system, adding a DNA construct encoding Rv0919 blocked synthesis, although only when acetyl coenzyme A was added (Fig. 1D). This suggests that Rv0919 uses acetyl coenzyme A as an acetyl group donor, which has been seen with other TacAT systems (14). A construct encoding Rv0919 with an active site mutation homologous to one identified in Salmonella (Y138F in M. tuberculosis) partially abrogated protein synthesis (Fig. 1E) (13). These results suggest that Rv0919 inhibits growth by acetylating a component of the protein synthesis apparatus.

**FIG 2:**
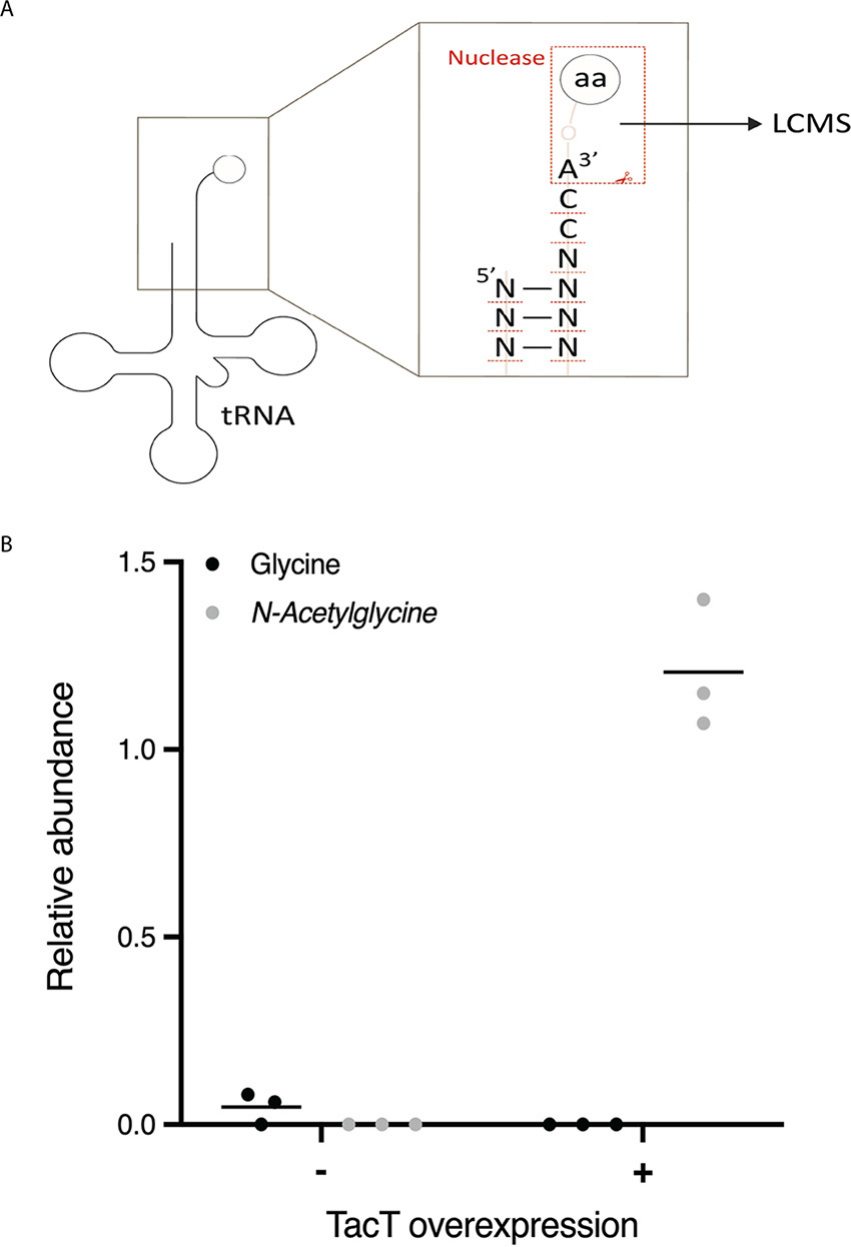
TacT acetylates glycyl-tRNA. M. smegmatis overexpressing M. tuberculosis *tacT* was grown to mid-log-phase and induced for 3 h. (A) Total RNA from triplicate cultures was collected along with an uninduced control for LCMS as described. (B) The relative abundance of unacetylated and *N*-acetylated glycyl-tRNA fragments is shown.

All other described TacAT-like systems encode a toxin that acetylates the amino acid on charged tRNA. Different organism toxins acetylate different tRNAs. For instance, in Salmonella, three different TacT-like toxins have been described. These block elongation by acetylating glycyl, isoleucyl-, leucyl-, and, to a lesser extent, other aminoacyl tRNAs *in vitro* (13) but solely glycyl-tRNA *in vivo* (in preparation). Meanwhile, in E. coli, the GNAT toxin AtaT was initially thought to block the initiation of protein synthesis by acetylating methionine on initiator fMet-tRNA (17) but was more recently reported to acetylate preferentially glycyl-tRNA alongside others (19). We hypothesized that M. tuberculosis TacT also acetylates charged tRNAs. To identify if any and which tRNA species might be affected by this enzyme, we purified total RNA from M. smegmatis overexpressing M. tuberculosis TacT and used liquid chromatography-coupled mass spectrometry (LCMS) to analyze tRNAs (Fig. 2A). A strong acetylation peak was detected for glycyl-tRNA (Fig. 2B; Table S1) but not for any other tRNAs, indicating that M. tuberculosis TacT specifically acetylates glycyl-tRNAs.

### Peptidyl tRNA hydrolase (Pth) reverses TacT-induced translation inhibition.

**FIG 3:**
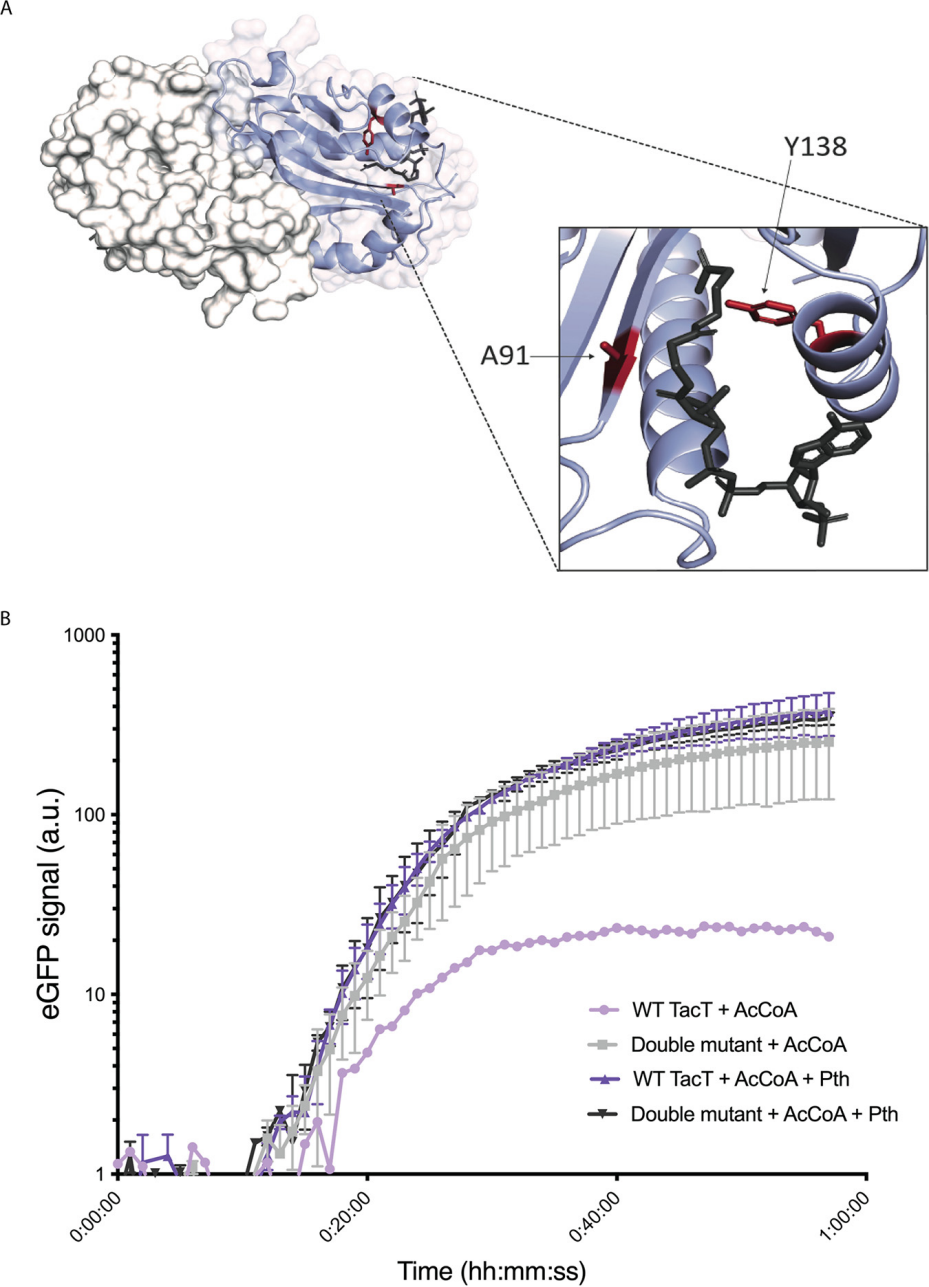
M. tuberculosis Pth detoxifies TacT. (A) Model of M. tuberculosis TacT dimer generated as described in Fig. 1, with one monomer showing mutations for catalytic inactivation (Y138F and A91P; red). Acetyl coenzyme A is shown in the TacT binding pocket and colored by element. (B) Cell-free protein synthesis reactions were set up as described in Fig. 1. Inactive TacT (“Double mutant”) was made using an Rv0919 expression construct containing both active side residue mutations Y138F and A91P. Purified M. tuberculosis peptidyl tRNA hydrolase (Pth) was added where indicated (8 μM), as was acetyl coenzyme A (AcCoA; 2 nM). In reactions without Pth, an equal volume of storage buffer was added. Protein synthesis was read out as eGFP synthesis and monitored spectrophotometrically at excitation of 488 nm and emission of 509 nm. Results are from technical duplicates.

Previous work has shown that the enzyme peptidyl tRNA hydrolase (Pth) detoxifies the effects of TacT acetylation by cleaving acetylated amino acids from corrupted tRNAs (14). To test whether M. tuberculosis Pth reverses TacT activity, we purified recombinant M. tuberculosis Pth and added it to our cell-free protein synthesis assay (Fig. S1). Indeed, purified Pth was sufficient to rescue GFP expression in the presence of active M. tuberculosis TacT and acetyl coenzyme A (Fig. 3B) but had no effects on translation in the presence of catalytically inactive TacT (Fig. 3A and B). Thus, M. tuberculosis Pth also cleaves N-acetylated aminoacyl-tRNA thereby counteracting the effect of the toxin.

### *tacAT* does not affect *pth* essentiality in M. tuberculosis.

**FIG 4:**
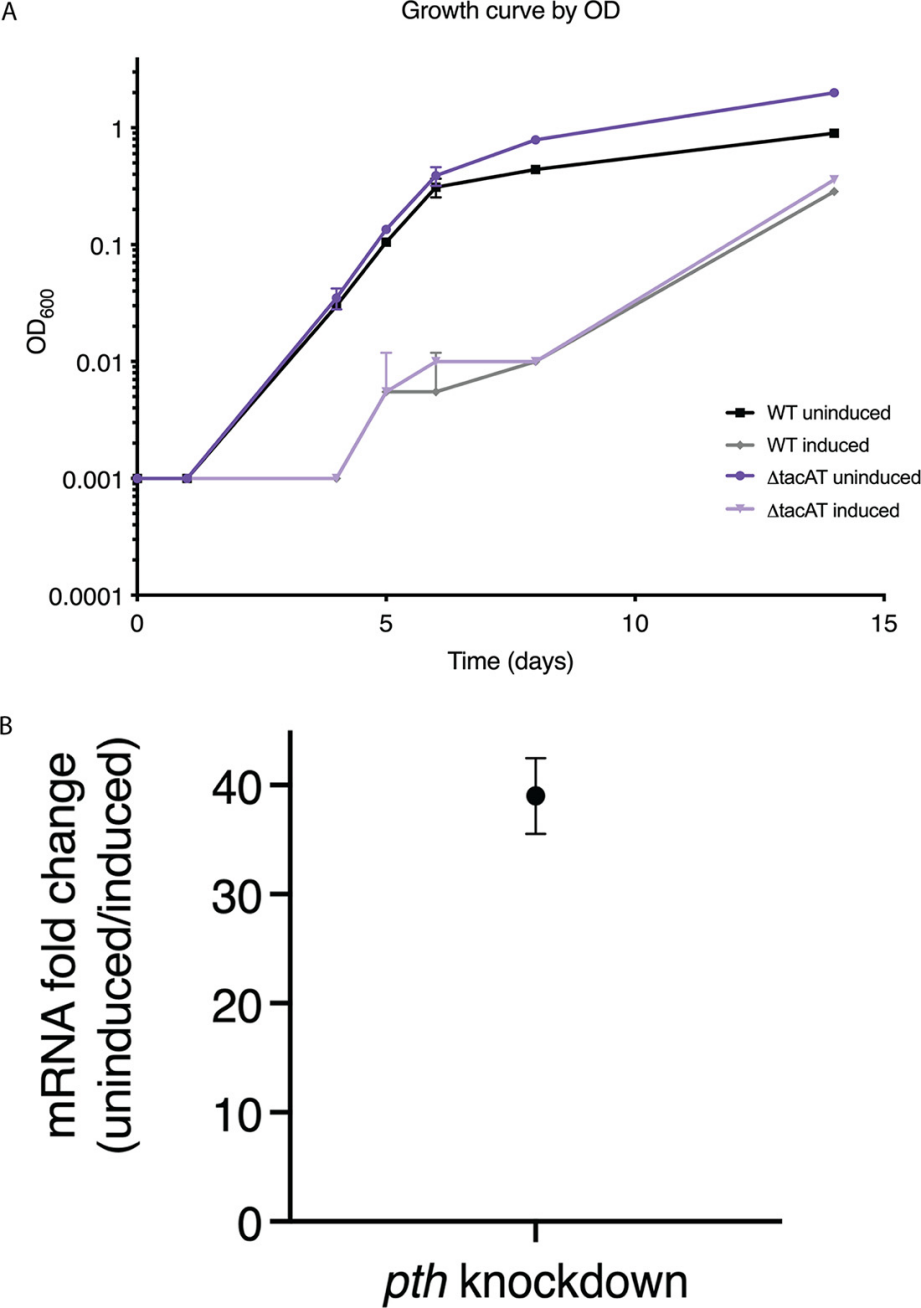
*pth* is required for normal growth of an M. tuberculosis *tacAT* knockout. (A) *MtbΔtacAT* and WT M. tuberculosis (H37Rv; parental strain) were transformed with *pth* knockdown constructs using mycobacterial CRISPR-interference (CRISPRi). Strains were diluted to an OD600 of 0.001 and either induced for *pth* knockdown (100 ng/mL aTC) or uninduced. Growth was measured by optical density at 600 nm (OD_600_). Results are from biological duplicates. (B) Changes in *pth* transcript levels during a knockdown, as measured by RT-qPCR. Relative fold change of each mRNA was quantified by normalization to levels of M. tuberculosis *sigA* transcript. Points represent the mean of three biological replicates, with error bars depicting standard deviation.

In addition to reversing the effects of TacT-like toxins, Pth’s primary known function is to cleave short peptides from peptidyl-tRNAs that are prematurely released from stalled ribosomes (23, 24). Similar to other bacteria, transposon sequencing (TnSeq) data suggest that *pth* is essential in M. tuberculosis (20). We built M. tuberculosis *pth* transcriptional knockdowns using CRISPR interference (CRISPRi) (25). Cells induced for *pth* depletion showed a marked growth defect, confirming that Pth is required for normal growth (Fig. 4).

Given TacAT’s connection to this essential enzyme, we assessed whether it contributes to *pth* essentiality in M. tuberculosis. Transposon sequencing data indicate that the TacAT-encoding operon Rv0918-0919 is nonessential for M. tuberculosis growth *in vitro*, and we have identified premature stop mutations in this TA system in clinical isolates, suggesting it is also dispensable *in vivo* (Table S2) (20). We built in-frame deletions of *tacAT* in M. tuberculosis and used CRISPRi to knock down *pth* in this strain. In the absence of *tacAT*, *pth* knockdowns still failed to grow normally *in vitro*, suggesting that while the *tacAT* operon is dispensable, *pth* essentiality is unaffected by the absence of this TA system (Fig. 4). We also performed LCMS on M. tuberculosis induced or uninduced for *pth* knockdown and were unable to detect glycyl-tRNA acetylation in either strain grown (Fig. S2). Thus, growth defects of a *pth* knockdown *in vitro* are not a result of the accumulation of acetylated glycyl-tRNAs.

## DISCUSSION

Toxin-antitoxin (TA) systems have been identified in most bacterial genomes and have been implicated in a variety of physiological functions ranging from phage protection and plasmid maintenance to pathogenesis and the general stress response. Interestingly, Mycobacterium tuberculosis encodes one of the largest repertoires of TA systems in bacteria, yet plasmids are absent from this organism (26). Furthermore, the role of TA systems in M. tuberculosis against bacteriophages is still under study (27). It is tempting to speculate that M. tuberculosis’s broad TA system toolkit serves as a growth regulator during human infection. However, experimental evidence for this is lacking, largely due to the difficulties of systematically deleting many genes simultaneously in M. tuberculosis and overlapping mechanisms of action that make it difficult to directly measure the activity of individual toxins. Some studies have examined the roles of individual TA systems in M. tuberculosis using genetic deletions and overexpression systems and linked the activity of some toxins to pathogenesis (10, 28, 29). Nonetheless, the level and spectrum of TA system involvement during M. tuberculosis infection remain unresolved.

Here, we have identified and characterized a TA system in M. tuberculosis whose mechanism of action is distinct from the other known TA systems in this organism. While most toxins in M. tuberculosis are ribonucleases, TacT instead blocks growth by acetylating charged tRNAs. This activity can be detected using liquid chromatography-coupled mass spectrometry (LCMS), making it an appealing TA system to study in its native form. We have shown that M. tuberculosis TacT acetylates glycyl tRNAs using an overexpression construct in M. smegmatis but have been unable to detect this modification in wild-type M. tuberculosis. Future work that increases the sensitivity and throughput of LCMS-based or other forms of detection for tRNA acetylation will allow researchers to probe the effects of various physiological conditions on this tRNA modification and identify conditions during which TacT is activated in M. tuberculosis and other bacteria containing homologous TA systems.

Recent work using genome sequences from clinical isolates of M. tuberculosis has shed light on the selective pressures imposed on M. tuberculosis’s genome during human infection (30, 31). The essentiality of a gene is correlated with its level of tolerance for nonsynonymous mutations (32). We have found that the TacAT operon in M. tuberculosis is dispensable *in vitro*, and clinical genomic data support that this operon is also dispensable *in vivo*, given many nonsynonymous mutations – including premature stop codons that have accumulated in clinical strains.

The other unique aspect of TacAT is its mechanistic connection to the essential enzyme peptidyl tRNA hydrolase (Pth), which reverses TacT-induced aminoacyl tRNA acetylation. Pth is ubiquitous and thought to be essential across bacteria. The three critical active site residues His22, Asp95, and Asn116 are universally conserved (33). Archaea, meanwhile, encode a conserved functional homolog, *pth2*, which does not share significant sequence similarity to bacterial *pth* (33). Most eukaryotes contain both *pth* and *pth2* genes, though these enzymes are individually nonessential. Interestingly, structural studies have found that mycobacterial Pth is divergent from other bacterial Pth in several regions (34, 35). Because of its essentiality in bacteria and unique structure in mycobacteria, in addition to the vulnerability of translation rescue systems in M. tuberculosis, Pth has been proposed as an intriguing drug target in difficult-to-treat organisms like M. tuberculosis (34, 36, 37). Understanding the critical functions of Pth is important from a drug development perspective, especially when considering potential sources for antibiotic resistance. For instance, if TacT activity were a significant source for *pth* essentiality in M. tuberculosis, then inhibitors targeting Pth would lose efficacy in clinical isolates with a disrupted *tacAT* operon. Our work has shown that M. tuberculosis TacT’s connection to Pth is not the source of *pth* essentiality. This bodes well for studies of Pth as an antibiotic target because mutations inactivating *tacAT* have already been identified in clinical isolates. Finally, biochemical assays to assess Pth inhibitors can exploit the relationship between Pth and TacT using *in vitro* protein synthesis kits by screening for loss of Pth-mediated detoxification.

## MATERIALS AND METHODS

### Bacterial strains and growth conditions.

M. tuberculosis and M. smegmatis strains were grown from frozen stocks into Middlebrook 7H9 medium supplemented with 0.2% glycerol, 0.05% Tween 80, and ADC (5g/L bovine serum albumin, 2g/L dextrose, 3 μg/mL catalase). Cultures were incubated at 37°C. Antibiotics or inducing agents were used when needed at the following concentrations in both M. tuberculosis and M. smegmatis: kanamycin (25 μg/mL), anhydrous tetracycline (aTC; 100 ng/mL), hygromycin (50 μg/mL), and nourseothricin (20 μg/mL). Transformed M. tuberculosis and M. smegmatis strains were plated onto 7H10 agar plates with the appropriate antibiotic(s). Strains were grown to mid-log-phase for all experiments unless otherwise specified (optical density at 600 nm (OD_600_) 0.4 to 0.6). E. coli strains for cloning or protein purification were grown in LB broth or on LB agar with appropriate antibiotics at the following concentrations: kanamycin (50 μg/mL), zeocin (50 μg/mL), and nourseothricin (40 μg/mL). The induction time for *pth* depletion in M. tuberculosis was 4 days. Induction for *tacT* overexpression in M. smegmatis was 3 h.

### Bacterial strain construction.

Table S3 depicts the strains, plasmids, primers, and recombinant DNA used for this study. Plasmids were built by restriction digest of a parental vector and inserts were prepared either by restriction enzyme cloning or Gibson assembly (38) using 40 bp overhangs, as specified in Table S3. Plasmids were isolated from E. coli and confirmed via Sanger sequencing carried out by Genewiz, LLC (MA, USA).

### Deletion mutants.

The knockout strain Δ*tacAT*::zeo (zeocin) was built using double-stranded recombineering in the parental M. tuberculosis strain H37Rv. A linear dsDNA fragment was constructed using stitch PCR with the primers listed in Table S3, which consisted of a 500 bp region upstream of the *tacAT* operon (Rv0918-019), 500 bp downstream region, and a *lox-*zeo*-lox* fragment. This cassette was transformed into an H37Rv recombineering strain as described (39) and plated on 7H10 + zeocin plates.

### *tacAT* and *tacT* alleles.

Plasmid FT2, used for inducible *tact* overexpression in M. smegmatis, was generated using a parental vector (CT16) that integrates into the L5 mycobacterial phage site. This plasmid also encodes kanamycin resistance and contains both the tet promoter (directly upstream of *tacT*) and the tet repressor. CT16 was digested with ClaI and XbaI (New England Biolabs). *tacT* (Rv0919) was PCR-amplified and ligated into the plasmid using restriction cloning. Plasmid FT3, used for *tacAT* overexpression, was generated by placing *tacAT* together under the constitutive UV15 promoter in a parental vector (CT250) which was digested with NdeI and HindIII (New England Biolabs). The *tacAT* operon Rv0918-0919 was ligated to the plasmid using Gibson cloning.

### Pth-knockdown constructs.

Transcriptional knockdown of *pth* was accomplished using mycobacterial CRISPRi-interference (CRISPRi). Knockdown constructs were built as previously described (25) by annealing oligonucleotides for *pth* and ligating them into a linearized BsmBI-digested plasmid (CT 296; a gift from Jeremy Rock) that contains mycobacterial CRISPRi. The knockdown vector FT110 was transformed in both H37Rv wild type (WT) and ΔtacAT::ZeoR.

### Purification of M. tuberculosis Pth.

M. tuberculosis *pth* (Rv1014c) was cloned with a C-terminal 6× His tag and expressed from pET28a in BL21-CodonPlus (DE3)-RP E. coli under conditions similar to those previously described (40). One liter of log-phase culture (OD_600_ ~0.7) was induced with 1 mM isopropyl β-D-1-thiogalactopyranoside (IPTG) for 4 h at 37°C. Cells were harvested at 6,000g for 15 min, and the resulting pellet was frozen at −80°C. The pellet was thawed with a stir-bar at 4°C in lysis buffer containing 50 mM Tris HCl pH 7.5, 300 mM NaCl, 10% glycerol, DNase powder, 1 tablet complete EDTA-free protease inhibitor, and 2 mM 2-mercaptoethanol (BME), and cells were lysed using a French press. The lysate was clarified by spinning at 30,000g for 30 min and brought up to 20 mM imidazole pH 7.5. His-tagged Pth was then extracted via batch binding 2.5 mL equilibrated Ni-NTA beads incubated with lysate for 1 h at 4°C. Beads were collected and washed with 20 mL lysis buffer containing 2 mM BME. A second wash included 20 mL lysis buffer with 20 mM imidazole and 2 mM BME, followed by 5 mL of lysis buffer with 30 mM imidazole, and a final wash with 5 mL lysis buffer containing 40 mM imidazole and 2 mM BME. Samples were eluted with lysis buffer containing 200 mM imidazole pH 7.5 in 750 μL fractions and analyzed via SDS-PAGE (Fig. S1, left). The cleanest elution fractions (4 to 6 and 7 to 9) were desalted into lysis buffer containing BME, concentrated with a 10 kDa MWCO amicon ultra 4 spin column to 1 mL, and further purified by FPLC via gel filtration chromatography with a Superdex 75 Increase 10/300 GL column in buffer containing (25 mM Tris-HCl pH 7.5, 150 mM NaCl, 2 mM BME). Fractions were analyzed via SDS-PAGE (Fig. S1, right) and fractions 2d2-2d3 (at an elution volume of ~13 mL) were pooled and brought up to 5% glycerol with 2 mM fresh BME. Nanodrop readings suggested that other fractions containing what appeared to be pure Pth were contaminated by unknown nucleic acid species. Nucleic acid-free protein (fractions 2d2-2d3) was aliquoted into 10 μL aliquots, flash-frozen with liquid nitrogen, and stored at −80°C. Pth protein concentration was calculated using a Coomassie Plus (Bradford) Assay (Pierce).

### *In vitro* translation.

To assess the effect of TacT on translation, *in vitro* translation reactions were prepared with purified *tacT* DNA (WT, Y138F, or Y138F/A91P), 2 nM acetyl coenzyme A, and purified Pth. A master mix of purified eGFP DNA (200 ng per reaction), *tact* DNA (180 ng per reaction), and PURExpress (New England Biolabs) components were prepared in triplicate reactions with 8 μM Pth and 2 nM acetyl coenzyme A. When no Pth was added, an equal volume of storage buffer was used in place of protein. When no acetyl coenzyme A was added, an equal volume of water was added. Reactions were carried out in 12 μL in a black Costar 384-well plate for 2 h at 37°C, and eGFP fluorescence (excitation = 488 nm and emission = 509 nm) was measured over time on a SpectraMax M2 microplate reader.

### mRNA quantification.

Ten milliliters of M. tuberculosis cultures were harvested at 4,000 rpm for 10 min and pellets were resuspended in 1 mL TRIzol reagent (ThermoFisher Scientific). Samples were lysed by bead beating. Purified DNase-treated RNA was used as the template for cDNA synthesis, following the manufacturer’s instructions with Superscript IV (Life Technologies). RNA was removed using RNase A (ThermoFisher Scientific) and cDNA was cleaned up by column purification (Zymo Research). qPCR was performed using iTaq Universal SYBR Green Supermix (Bio-Rad). mRNA fold change was calculated using the ΔΔCt method, where *pth* transcript level was normalized by *sigA* level under each condition.

### Liquid chromatography-coupled mass spectrometry.

Purified RNA (30 to 50 μg) was spiked with 1 μM ^15^N-AMP and incubated with 1U Nuclease P1 in 10 mM ammonium acetate for 30 min at 25°C (Fig. 2 and Fig. S2). Alternatively, purified RNA was incubated with 25 μg purified Pth in buffer (10 mM Tris-acetate, 10 mM magnesium acetate, 20 mM ammonium acetate pH 8) for 1 h at 37°C. Processed RNA samples were diluted 1:3 with acetonitrile + 0.2% vol/vol acetic acid, centrifuged for 10 min at 21,000 × *g*, room temperature to remove any precipitate, and transferred to glass microvials. Samples were analyzed on a Thermo Ultimate 3000 LC coupled with a Q-Exactive Plus mass spectrometer in both positive and negative ion modes. Five microliters of each sample were injected on a Zic-pHILIC Column (150 × 2.1 mm, 5 μm particles, EMD Millipore). The mobile phases are (A) 20 mM ammonium carbonate in 0.1% ammonium hydroxide and (B) acetonitrile 97% in water. The gradient conditions were as follows: 100% B at 0 min, 40% B at 20 min, 0% B at 30 min for 5 min, then back to 100% B in 5 min, followed by 10 min of re-equilibration. A constant flow rate of 0.200 L/minute was used. The mass spectrometer was calibrated immediately before use. Data were analyzed using Thermo Xcalibur 3.0 with ICIS automated peak integration (Default settings: Smoothing Points = 9; Baseline Window = 40; Area Noise Factor = 2; Peak Noise Factor = 10) followed by manual data curation. In the analysis of NP1 treated samples (Fig. 2 and Fig. S2), peak areas were normalized relative to the peak area of the ^15^N-AMP spike-in. To distinguish between isobaric tRNA fragments derived from *N*-acetylserine and glutamic acid ([M+H]+ ion *m/z* = 476.1057), RNA samples instead treated with Pth (to cleave *N*-acetyl amino acids from tRNAs) were compared by LC/MS to purified standards of each amino acid, allowing for identification of peaks discriminated by retention time (Fig. S3). These data indicate that glutamic acid, and not *N*-acetylserine, contributes the entirety of the MS signal detected for ions with an *m/z* of 147.0532 and that TacT does not acetylate seryl-tRNA.

### Whole-genome sequencing analysis of clinical isolates.

Whole-genome sequences of 55778 M. tuberculosis isolates were obtained from 211 BioProjects under the following accession codes: ERP001037, ERP002611, ERP008770, PRJDB10607, PRJDB3875, PRJDB6149, PRJDB7006, PRJDB8544, PRJDB8553, PRJEB10385, PRJEB10533, PRJEB10577, PRJEB10950, PRJEB11460, PRJEB11653, PRJEB11778, PRJEB12011, PRJEB12179, PRJEB12184, PRJEB12764, PRJEB13325, PRJEB13764, PRJEB13960, PRJEB14199, PRJEB15076, PRJEB15382, PRJEB15857, PRJEB18529, PRJEB20214, PRJEB21685, PRJEB21888, PRJEB21922, PRJEB23245, PRJEB23495, PRJEB2358, PRJEB23648, PRJEB23664, PRJEB23996, PRJEB24463, PRJEB25506, PRJEB25543, PRJEB25592, PRJEB25814, PRJEB25968, PRJEB25971, PRJEB25972, PRJEB25991, PRJEB25997, PRJEB25998, PRJEB25999, PRJEB26000, PRJEB26001, PRJEB26002, PRJEB27244, PRJEB27354, PRJEB27366, PRJEB27446, PRJEB27847, PRJEB2794, PRJEB28497, PRJEB28842, PRJEB29199, PRJEB29276, PRJEB29408, PRJEB29435, PRJEB29446, PRJEB29604, PRJEB30463, PRJEB30782, PRJEB30933, PRJEB31023, PRJEB31905, PRJEB32037, PRJEB32234, PRJEB32341, PRJEB32589, PRJEB32684, PRJEB32773, PRJEB33896, PRJEB35201, PRJEB39699, PRJEB40777, PRJEB5162, PRJEB5280, PRJEB5899, PRJEB5925, PRJEB6273, PRJEB6717, PRJEB6945, PRJEB7056, PRJEB7281, PRJEB7669, PRJEB7727, PRJEB7798, PRJEB8311, PRJEB8432, PRJEB8689, PRJEB9003, PRJEB9201, PRJEB9206, PRJEB9308, PRJEB9545, PRJEB9680, PRJEB9709, PRJEB9976, PRJNA200335, PRJNA217391, PRJNA219826, PRJNA220218, PRJNA229360, PRJNA233386, PRJNA235852, PRJNA237443, PRJNA244659, PRJNA254678, PRJNA259657, PRJNA268900, PRJNA270137, PRJNA282721, PRJNA287858, PRJNA295328, PRJNA300846, PRJNA302362, PRJNA305488, PRJNA306588, PRJNA308536, PRJNA318002, PRJNA352769, PRJNA353873, PRJNA354716, PRJNA355614, PRJNA356104, PRJNA361483, PRJNA369219, PRJNA376471, PRJNA377769, PRJNA379070, PRJNA384604, PRJNA384765, PRJNA384815, PRJNA385247, PRJNA388806, PRJNA390065, PRJNA390291, PRJNA390471, PRJNA393378, PRJNA393923, PRJNA393924, PRJNA401368, PRJNA401515, PRJNA407704, PRJNA413593, PRJNA414758, PRJNA419964, PRJNA421323, PRJNA421446, PRJNA428596, PRJNA429460, PRJNA430531, PRJNA431049, PRJNA436223, PRJNA436997, PRJNA438921, PRJNA448595, PRJNA453687, PRJNA454477, PRJNA475130, PRJNA475771, PRJNA480117, PRJNA480888, PRJNA481625, PRJNA481638, PRJNA482095, PRJNA482716, PRJNA482865, PRJNA486713, PRJNA488343, PRJNA488426, PRJNA492975, PRJNA506272, PRJNA509547, PRJNA512266, PRJNA522942, PRJNA523164, PRJNA523499, PRJNA524863, PRJNA526078, PRJNA533314, PRJNA540911, PRJNA549270, PRJNA559678, PRJNA566379, PRJNA573497, PRJNA578162, PRJNA586859, PRJNA587747, PRJNA589048, PRJNA591498, PRJNA595834, PRJNA598949, PRJNA598981, PRJNA608715, PRJNA632617, PRJNA663350, PRJNA678116, PRJNA679443, PRJNA683067, PRJNA684613, PRJNA688213, SRP018402. The Sickle tool was used for trimming whole-genome sequencing data (41). Sequencing reads with Phred base quality scores above 20 and read lengths longer than 30 were kept for analysis. The inferred ancestral genome of the most recent common ancestor of the MTBC was used as the reference template for read mapping (42). Sequencing reads were mapped to the reference genome using Bowtie 2 (version 2.2.9) (43). SAMtools (v1.3.1) was used for SNP calling with mapping quality greater than 30. Fixed mutations (frequency ≥ 75%) were identified using VarScan (v2.3.9) with at least 10 supporting reads and the strand bias filter option on. SNPs in repetitive regions of the genome (e.g., PPE/PE-PGRS family genes, phage sequences, insertion, or mobile genetic elements) were excluded (44, 45).

### Data availability.

The data that support these findings are available from the corresponding author upon reasonable request.
