# Supplementary material for: A tRNA-Acetylating Toxin and Detoxifying Enzyme in Mycobacterium tuberculosis
Source: Microbiol Spectr. 2022 May 31;10(3):e00580-22. doi: 10.1128/spectrum.00580-22 (PMC9241777; doi:10.1128/spectrum.00580-22)
Supplement: Supplemental file 4 — Fig. S1 to S3. Download spectrum.00580-22-s0003.pdf, PDF file, 0.2 MB [file spectrum.00580-22-s0003.pdf]

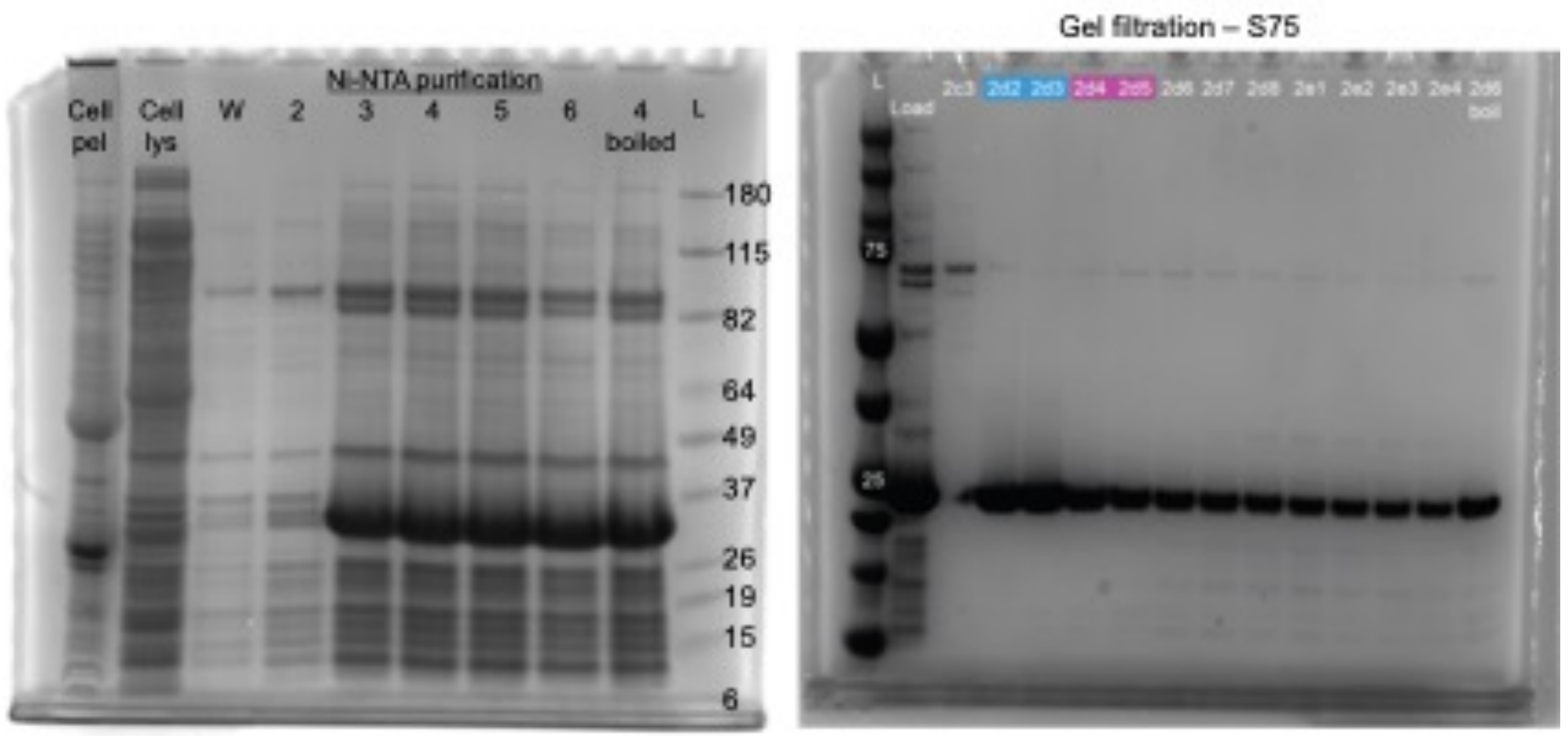

Supplementary Figure 1. *Mtb* Pth purification gels.

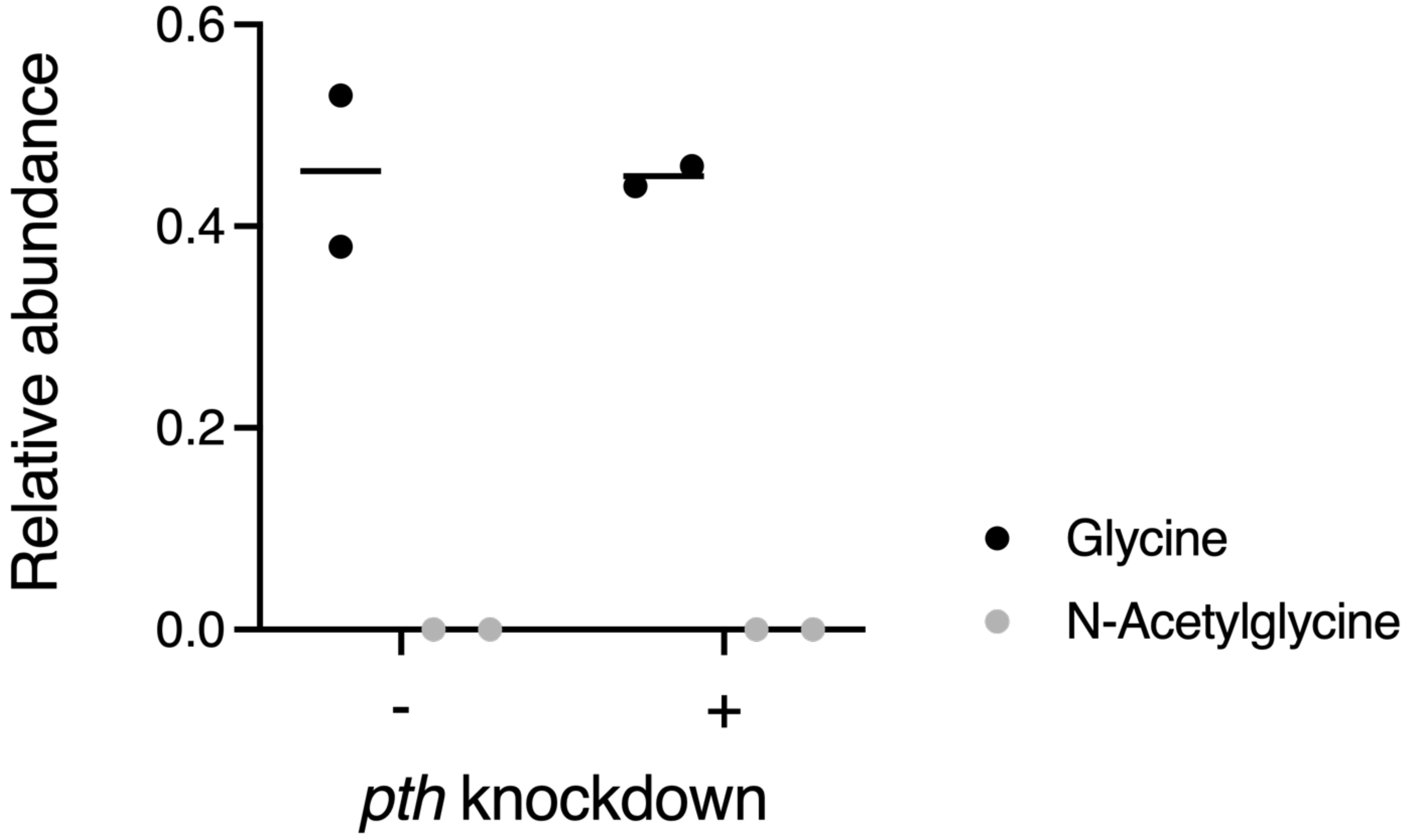

Supplementary Figure 2: TacT is not active in normal *Mtb* laboratory growth conditions. Wild-type *Mtb* was induced for *pth* depletion and incubated for 4 days. Total RNA from duplicate cultures was collected along with an uninduced control for liquid chromatography-mass spectrometry analysis as described. The relative abundance of unacetylated and N-acetylated glycyl-tRNA fragments are shown.

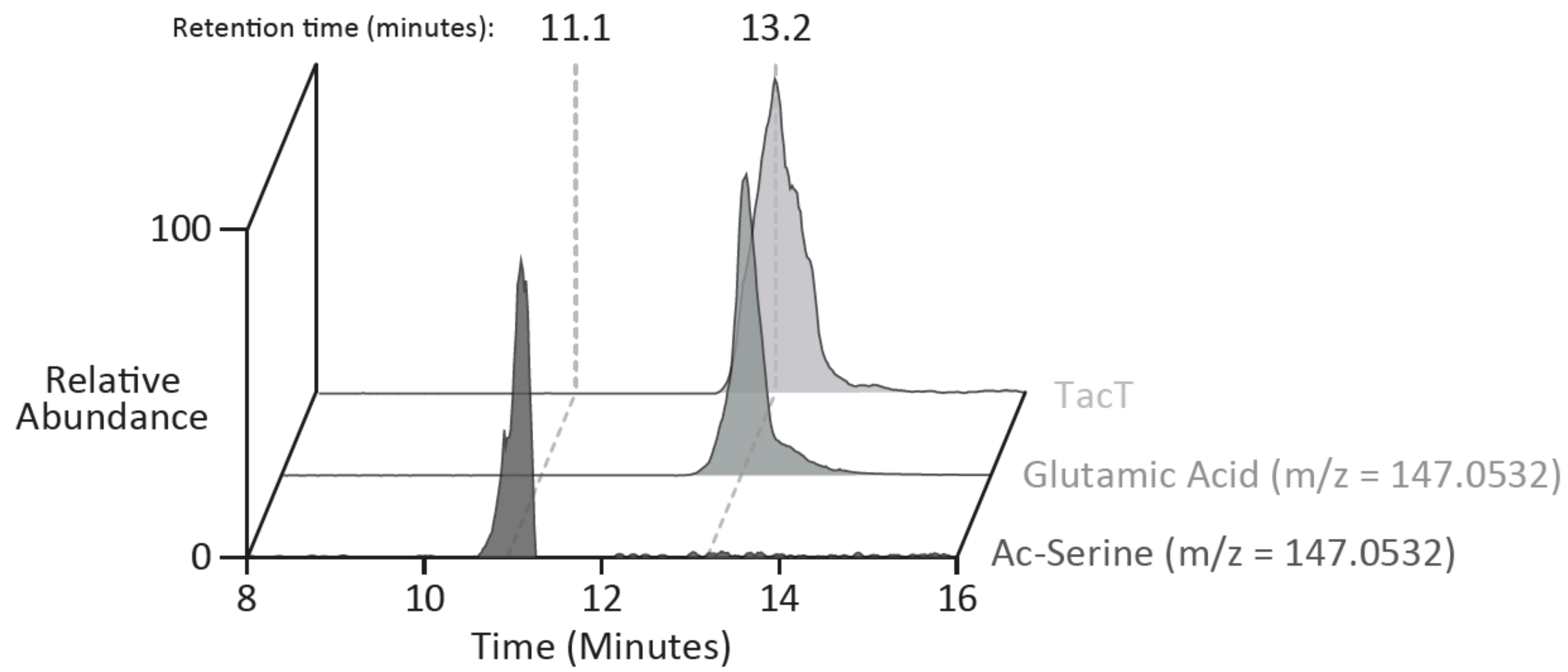

Supplementary Figure 3: Distinction between acetylated seryl-tRNA and glutamine tRNA. RNA samples treated with Pth were compared to purified standards of each amino acid as described in the Materials and Methods.
